# *N*′-(2-Hydr­oxy-3-methoxy­benzyl­idene)-1,3-benzodioxole-5-carbohydrazide monohydrate

**DOI:** 10.1107/S1600536808040117

**Published:** 2008-12-06

**Authors:** Chun-Lin Du

**Affiliations:** a College of Chemical Engineering & Materials Science, Liaodong University, Dandong 118003, People’s Republic of China

## Abstract

Single crystals of the title compound, C_16_H_14_N_2_O_5_·H_2_O, were obtained from a condensation reaction of 1,3-benzodioxole-5-carbohydrazide and 3-methoxy­salicylaldehyde in a 95% ethanol solution. The asymmetric unit consists of a Schiff base mol­ecule, which assumes an *E* configuration with respect to the C=N bond, and a water mol­ecule of crystallization. The dihedral angle between the two substituted benzene rings is 12.7 (2)°. In the crystal structure, mol­ecules are linked through inter­molecular N—H⋯O and O—H⋯O hydrogen bonds, forming layers parallel to the *bc* plane.

## Related literature

For the biological properties of hydrazones, see: Bedia *et al.* (2006 ▶); Rollas *et al.* (2002 ▶); Okabe *et al.* (1993 ▶). For bond-length data, see: Allen *et al.* (1987 ▶). For related structures, see: Fun *et al.* (2008 ▶); Qu *et al.* (2008 ▶); Shan *et al.* (2008 ▶); Yehye *et al.* (2008 ▶).
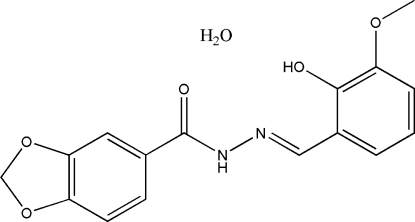


## Experimental

### 

#### Crystal data

C_16_H_14_N_2_O_5_·H_2_O
*M* *_r_* = 332.31
Orthorhombic, 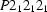

*a* = 4.792 (2) Å
*b* = 12.916 (3) Å
*c* = 24.002 (6) Å
*V* = 1485.6 (7) Å^3^
*Z* = 4
Mo *K*α radiation
μ = 0.12 mm^−1^
*T* = 298 (2) K
0.23 × 0.20 × 0.20 mm

#### Data collection

Bruker SMART 1K CCD area-detector diffractometer
Absorption correction: multi-scan (*SADABS*; Sheldrick, 1996 ▶) *T*_min_ = 0.974, *T*_max_ = 0.977
8595 measured reflections
1907 independent reflections
1639 reflections with *I* > 2σ(*I*)
*R*_int_ = 0.030

#### Refinement

*R*[*F* ^2^ > 2σ(*F* ^2^)] = 0.032
*wR*(*F* ^2^) = 0.076
*S* = 1.05
1907 reflections
228 parameters
4 restraints
H atoms treated by a mixture of independent and constrained refinement
Δρ_max_ = 0.13 e Å^−3^
Δρ_min_ = −0.13 e Å^−3^

### 

Data collection: *SMART* (Bruker, 2002 ▶); cell refinement: *SAINT* (Bruker, 2002 ▶); data reduction: *SAINT*; program(s) used to solve structure: *SHELXS97* (Sheldrick, 2008 ▶); program(s) used to refine structure: *SHELXL97* (Sheldrick, 2008 ▶); molecular graphics: *SHELXTL* (Sheldrick, 2008 ▶); software used to prepare material for publication: *SHELXTL*.

## Supplementary Material

Crystal structure: contains datablocks global, I. DOI: 10.1107/S1600536808040117/sj2559sup1.cif

Structure factors: contains datablocks I. DOI: 10.1107/S1600536808040117/sj2559Isup2.hkl

Additional supplementary materials:  crystallographic information; 3D view; checkCIF report

## Figures and Tables

**Table 1:** Hydrogen-bond geometry (Å, °)

*D*—H⋯*A*	*D*—H	H⋯*A*	*D*⋯*A*	*D*—H⋯*A*
O1—H1⋯N1	0.82	2.04	2.743 (2)	143
O1—H1⋯O6	0.82	2.56	3.001 (2)	115
N2—H2⋯O6^i^	0.899 (10)	2.075 (11)	2.962 (2)	168 (3)
O6—H6*A*⋯O2	0.857 (10)	1.880 (12)	2.728 (2)	170 (3)
O6—H6*B*⋯O1^ii^	0.848 (10)	2.269 (16)	3.043 (2)	152 (2)
O6—H6*B*⋯O3^ii^	0.848 (10)	2.538 (19)	3.226 (2)	139 (2)

Symmetry codes: (i) 
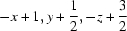
; (ii) 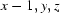.

**Fig. 1.**
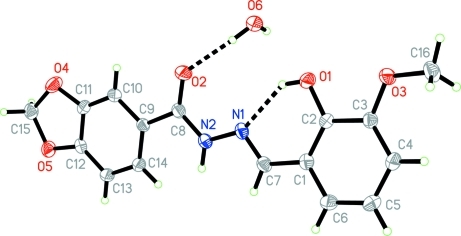
The structure of the title compound, showing 30% probability displacement ellipsoids and the atom-numbering scheme.

**Fig. 2.**
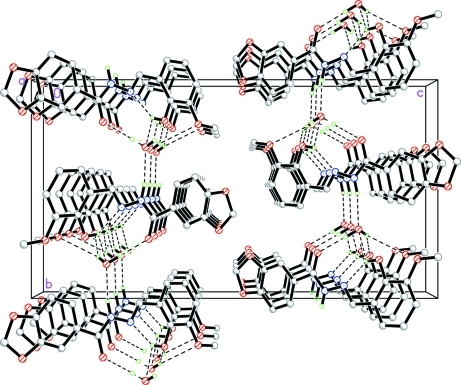
The crystal packing of the title compound, viewed down the a axis.

## supplementary crystallographic information

### Comment

Hydrazone compounds, derived from the condensation reactions of aldehydes with hydrazides, show interesting biological properties (Okabe *et al.*, 1993; Bedia *et al.*, 2006; Rollas *et al.*, 2002). Recently, a large number of hydrazone derivatives have been reported (Shan *et al.*, 2008; Fun *et al.*, 2008; Qu *et al.*, 2008; Yehye *et al.*, 2008). We report here the structure of a new hydrazone compound, I, Fig. 1, with a Schiff base molecule, which assumes an *E* configuration with respect to the C═N bond  and a water molecule in the asymmetric unit. The dihedral angle between the two substituted benzene rings is 12.7 (2)°. All the bond lengths are within normal ranges (Allen *et al.*, 1987).

In the crystal structure, molecules are linked through intermolecular N—H···O and O—H···O hydrogen bonds (Table 1), forming layers parallel to the *bc* direction (Fig. 2).

### Experimental

The title compound was prepared by Schiff base condensation reaction of 1,3-benzodioxole-5-carbohydrazide (1.0 mmol) and 3-methoxysalicylaldehyde (1.0 mmol) in a 95% ethanol solution (50 ml). Needle colorless crystals were formed by gradual evaporation of the solution in air for a few days.

### Refinement

The imino H atom was located in a difference map and refined with a N–H distance restraint of 0.90 (1) Å. The water H atoms were also located in a difference map and refined with O–H and H···H distances restraints of 0.85 (1) and 1.37 (2) Å, respectively. The other H atoms were positioned geometrically [C–H = 0.93–0.97 Å] and refined using a riding model, with *U*_iso_(H) = 1.2*U*_eq_(C) and 1.5*U*_eq_(C_methyl_). A rotating group model was used for the methyl group. In the absence of significant anomalous scattering effects, 1034 Friedel pairs were merged.

### Crystal data

**Table:**

C_16_H_14_N_2_O_5_·H_2_O	*F*(000) = 696
*M**_r_* = 332.31	*D*_x_ = 1.486 Mg m^−^^3^
Orthorhombic, *P*2_1_2_1_2_1_	Mo *K*α radiation, λ = 0.71073 Å
Hall symbol: P 2ac 2ab	Cell parameters from 3222 reflections
*a* = 4.792 (2) Å	θ = 2.4–25.6°
*b* = 12.916 (3) Å	µ = 0.12 mm^−^^1^
*c* = 24.002 (6) Å	*T* = 298 K
*V* = 1485.6 (7) Å^3^	Cut from needle, colorless
*Z* = 4	0.23 × 0.20 × 0.20 mm

### Data collection

**Table:**

Bruker SMART 1K CCD area-detector diffractometer	1907 independent reflections
Radiation source: fine-focus sealed tube	1639 reflections with *I* > 2σ(*I*)
graphite	*R*_int_ = 0.030
ω scans	θ_max_ = 27.0°, θ_min_ = 1.7°
Absorption correction: multi-scan (*SADABS*; Sheldrick, 1996)	*h* = −6→6
*T*_min_ = 0.974, *T*_max_ = 0.977	*k* = −16→13
8595 measured reflections	*l* = −30→28

### Refinement

**Table:**

Refinement on *F*^2^	Primary atom site location: structure-invariant direct methods
Least-squares matrix: full	Secondary atom site location: difference Fourier map
*R*[*F*^2^ > 2σ(*F*^2^)] = 0.032	Hydrogen site location: inferred from neighbouring sites
*wR*(*F*^2^) = 0.076	H atoms treated by a mixture of independent and constrained refinement
*S* = 1.05	*w* = 1/[σ^2^(*F*_o_^2^) + (0.0385*P*)^2^ + 0.1335*P*] where *P* = (*F*_o_^2^ + 2*F*_c_^2^)/3
1907 reflections	(Δ/σ)_max_ < 0.001
228 parameters	Δρ_max_ = 0.13 e Å^−^^3^
4 restraints	Δρ_min_ = −0.13 e Å^−^^3^

### Special details

**Table:**

Geometry. All e.s.d.'s (except the e.s.d. in the dihedral angle between two l.s. planes) are estimated using the full covariance matrix. The cell e.s.d.'s are taken into account individually in the estimation of e.s.d.'s in distances, angles and torsion angles; correlations between e.s.d.'s in cell parameters are only used when they are defined by crystal symmetry. An approximate (isotropic) treatment of cell e.s.d.'s is used for estimating e.s.d.'s involving l.s. planes.
Refinement. Refinement of *F*^2^ against ALL reflections. The weighted *R*-factor *wR* and goodness of fit *S* are based on *F*^2^, conventional *R*-factors *R* are based on *F*, with *F* set to zero for negative *F*^2^. The threshold expression of *F*^2^ > σ(*F*^2^) is used only for calculating *R*-factors(gt) *etc*. and is not relevant to the choice of reflections for refinement. *R*-factors based on *F*^2^ are statistically about twice as large as those based on *F*, and *R*- factors based on ALL data will be even larger.

### Fractional atomic coordinates and isotropic or equivalent isotropic displacement parameters (Å^2^)

**Table:**

	*x*	*y*	*z*	*U*_iso_*/*U*_eq_	
O1	0.9802 (3)	0.80320 (10)	0.83305 (6)	0.0447 (4)	
H1	0.8775	0.8121	0.8061	0.067*	
O2	0.3794 (4)	0.78499 (11)	0.69933 (6)	0.0563 (5)	
O3	1.3470 (3)	0.79317 (11)	0.91162 (6)	0.0489 (4)	
O4	−0.3191 (4)	0.81446 (12)	0.53841 (6)	0.0599 (5)	
O5	−0.3991 (3)	0.98897 (11)	0.52200 (6)	0.0490 (4)	
O6	0.4857 (4)	0.67908 (12)	0.79533 (6)	0.0528 (4)	
N1	0.7019 (4)	0.92306 (13)	0.75678 (6)	0.0368 (4)	
N2	0.5153 (4)	0.95059 (13)	0.71517 (7)	0.0374 (4)	
C1	1.0510 (4)	0.98797 (15)	0.82015 (7)	0.0349 (4)	
C2	1.1069 (4)	0.89400 (14)	0.84647 (8)	0.0330 (4)	
C3	1.3082 (4)	0.88967 (15)	0.88912 (8)	0.0360 (4)	
C4	1.4481 (5)	0.97831 (16)	0.90522 (8)	0.0415 (5)	
H4	1.5789	0.9754	0.9338	0.050*	
C5	1.3937 (5)	1.07197 (16)	0.87871 (8)	0.0449 (5)	
H5	1.4897	1.1314	0.8893	0.054*	
C6	1.1978 (5)	1.07680 (15)	0.83680 (8)	0.0418 (5)	
H6	1.1624	1.1397	0.8193	0.050*	
C7	0.8453 (5)	0.99888 (16)	0.77596 (8)	0.0388 (5)	
H7	0.8163	1.0643	0.7608	0.047*	
C8	0.3608 (5)	0.87806 (15)	0.68878 (7)	0.0371 (5)	
C9	0.1638 (5)	0.91647 (14)	0.64528 (7)	0.0330 (4)	
C10	0.0244 (5)	0.84061 (15)	0.61392 (8)	0.0383 (5)	
H10	0.0559	0.7705	0.6200	0.046*	
C11	−0.1597 (5)	0.87353 (15)	0.57404 (8)	0.0385 (5)	
C12	−0.2088 (5)	0.97742 (15)	0.56457 (7)	0.0359 (4)	
C13	−0.0799 (5)	1.05292 (15)	0.59480 (8)	0.0412 (5)	
H13	−0.1162	1.1227	0.5885	0.049*	
C14	0.1088 (5)	1.02087 (15)	0.63555 (8)	0.0386 (5)	
H14	0.2004	1.0705	0.6568	0.046*	
C15	−0.4686 (6)	0.88603 (18)	0.50433 (8)	0.0497 (6)	
H15A	−0.6678	0.8746	0.5080	0.060*	
H15B	−0.4179	0.8765	0.4655	0.060*	
C16	1.5697 (5)	0.78022 (19)	0.95022 (9)	0.0522 (6)	
H16A	1.5365	0.8226	0.9824	0.078*	
H16B	1.7421	0.8005	0.9330	0.078*	
H16C	1.5806	0.7089	0.9613	0.078*	
H2	0.499 (7)	1.0186 (9)	0.7078 (10)	0.080*	
H6A	0.474 (6)	0.7133 (18)	0.7649 (6)	0.080*	
H6B	0.366 (5)	0.706 (2)	0.8170 (8)	0.080*	

### Atomic displacement parameters (Å^2^)

**Table:**

	*U*^11^	*U*^22^	*U*^33^	*U*^12^	*U*^13^	*U*^23^
O1	0.0460 (9)	0.0381 (8)	0.0500 (9)	−0.0062 (7)	−0.0165 (7)	−0.0011 (7)
O2	0.0772 (12)	0.0351 (8)	0.0567 (9)	0.0061 (9)	−0.0217 (9)	0.0055 (7)
O3	0.0506 (10)	0.0414 (8)	0.0548 (8)	−0.0064 (8)	−0.0222 (8)	0.0105 (7)
O4	0.0706 (12)	0.0453 (8)	0.0638 (10)	0.0007 (9)	−0.0276 (10)	−0.0127 (8)
O5	0.0524 (10)	0.0516 (9)	0.0430 (8)	0.0055 (8)	−0.0141 (8)	−0.0009 (7)
O6	0.0684 (12)	0.0394 (8)	0.0505 (9)	0.0050 (9)	−0.0110 (9)	0.0016 (7)
N1	0.0366 (9)	0.0429 (9)	0.0308 (8)	0.0065 (8)	−0.0034 (8)	0.0020 (7)
N2	0.0394 (10)	0.0373 (9)	0.0356 (8)	0.0034 (8)	−0.0070 (8)	0.0044 (7)
C1	0.0326 (11)	0.0389 (10)	0.0333 (9)	0.0042 (9)	0.0022 (8)	−0.0020 (8)
C2	0.0319 (10)	0.0339 (9)	0.0332 (9)	−0.0007 (9)	−0.0005 (8)	−0.0039 (8)
C3	0.0357 (11)	0.0380 (10)	0.0344 (9)	−0.0002 (10)	−0.0025 (9)	0.0003 (8)
C4	0.0389 (12)	0.0462 (11)	0.0393 (10)	−0.0042 (10)	−0.0058 (10)	−0.0047 (9)
C5	0.0442 (13)	0.0380 (11)	0.0525 (12)	−0.0065 (10)	−0.0029 (11)	−0.0063 (9)
C6	0.0450 (13)	0.0335 (10)	0.0469 (11)	0.0031 (10)	0.0012 (11)	0.0001 (9)
C7	0.0418 (12)	0.0381 (10)	0.0365 (10)	0.0068 (11)	−0.0019 (9)	0.0038 (8)
C8	0.0424 (12)	0.0366 (10)	0.0323 (10)	0.0055 (10)	0.0005 (9)	0.0019 (8)
C9	0.0356 (11)	0.0333 (9)	0.0302 (9)	0.0016 (9)	0.0021 (8)	0.0020 (7)
C10	0.0446 (12)	0.0325 (10)	0.0377 (10)	0.0031 (10)	−0.0014 (10)	0.0007 (8)
C11	0.0419 (13)	0.0376 (10)	0.0360 (10)	−0.0013 (10)	−0.0009 (9)	−0.0064 (8)
C12	0.0357 (11)	0.0438 (11)	0.0283 (9)	0.0039 (9)	0.0002 (9)	0.0030 (8)
C13	0.0508 (13)	0.0314 (10)	0.0414 (10)	0.0050 (10)	−0.0056 (10)	0.0021 (9)
C14	0.0442 (12)	0.0345 (10)	0.0372 (10)	−0.0001 (9)	−0.0066 (10)	−0.0010 (8)
C15	0.0480 (14)	0.0602 (14)	0.0410 (11)	−0.0083 (13)	−0.0079 (11)	0.0006 (10)
C16	0.0456 (13)	0.0630 (14)	0.0478 (12)	−0.0021 (12)	−0.0128 (11)	0.0145 (11)

### Geometric parameters (Å, °)

**Table:**

O1—C2	1.359 (2)	C4—H4	0.9300
O1—H1	0.8200	C5—C6	1.377 (3)
O2—C8	1.232 (2)	C5—H5	0.9300
O3—C3	1.371 (2)	C6—H6	0.9300
O3—C16	1.423 (2)	C7—H7	0.9300
O4—C11	1.377 (2)	C8—C9	1.492 (3)
O4—C15	1.427 (3)	C9—C14	1.394 (3)
O5—C12	1.378 (2)	C9—C10	1.405 (3)
O5—C15	1.435 (3)	C10—C11	1.369 (3)
O6—H6A	0.857 (10)	C10—H10	0.9300
O6—H6B	0.848 (10)	C11—C12	1.381 (3)
N1—C7	1.282 (3)	C12—C13	1.363 (3)
N1—N2	1.387 (2)	C13—C14	1.395 (3)
N2—C8	1.352 (3)	C13—H13	0.9300
N2—H2	0.899 (10)	C14—H14	0.9300
C1—C2	1.394 (3)	C15—H15A	0.9700
C1—C6	1.404 (3)	C15—H15B	0.9700
C1—C7	1.455 (3)	C16—H16A	0.9600
C2—C3	1.408 (3)	C16—H16B	0.9600
C3—C4	1.382 (3)	C16—H16C	0.9600
C4—C5	1.392 (3)		
			
C2—O1—H1	109.5	N2—C8—C9	116.35 (16)
C3—O3—C16	117.72 (17)	C14—C9—C10	119.66 (18)
C11—O4—C15	105.99 (16)	C14—C9—C8	123.96 (18)
C12—O5—C15	105.81 (15)	C10—C9—C8	116.36 (17)
H6A—O6—H6B	105.5 (19)	C11—C10—C9	117.68 (18)
C7—N1—N2	114.10 (16)	C11—C10—H10	121.2
C8—N2—N1	120.86 (16)	C9—C10—H10	121.2
C8—N2—H2	122.6 (19)	C10—C11—O4	128.26 (18)
N1—N2—H2	116.6 (19)	C10—C11—C12	121.75 (19)
C2—C1—C6	119.08 (18)	O4—C11—C12	109.99 (18)
C2—C1—C7	123.01 (19)	C13—C12—O5	128.10 (17)
C6—C1—C7	117.90 (18)	C13—C12—C11	122.03 (18)
O1—C2—C1	123.91 (17)	O5—C12—C11	109.86 (17)
O1—C2—C3	116.37 (16)	C12—C13—C14	117.03 (17)
C1—C2—C3	119.72 (17)	C12—C13—H13	121.5
O3—C3—C4	125.24 (18)	C14—C13—H13	121.5
O3—C3—C2	114.56 (17)	C9—C14—C13	121.83 (18)
C4—C3—C2	120.20 (18)	C9—C14—H14	119.1
C3—C4—C5	120.09 (19)	C13—C14—H14	119.1
C3—C4—H4	120.0	O4—C15—O5	108.32 (17)
C5—C4—H4	120.0	O4—C15—H15A	110.0
C6—C5—C4	120.1 (2)	O5—C15—H15A	110.0
C6—C5—H5	120.0	O4—C15—H15B	110.0
C4—C5—H5	120.0	O5—C15—H15B	110.0
C5—C6—C1	120.83 (19)	H15A—C15—H15B	108.4
C5—C6—H6	119.6	O3—C16—H16A	109.5
C1—C6—H6	119.6	O3—C16—H16B	109.5
N1—C7—C1	123.44 (18)	H16A—C16—H16B	109.5
N1—C7—H7	118.3	O3—C16—H16C	109.5
C1—C7—H7	118.3	H16A—C16—H16C	109.5
O2—C8—N2	122.73 (19)	H16B—C16—H16C	109.5
O2—C8—C9	120.92 (19)		

### Hydrogen-bond geometry (Å, °)

**Table:**

*D*—H···*A*	*D*—H	H···*A*	*D*···*A*	*D*—H···*A*
O1—H1···N1	0.82	2.04	2.743 (2)	143
O1—H1···O6	0.82	2.56	3.001 (2)	115
N2—H2···O6^i^	0.90 (1)	2.08 (1)	2.962 (2)	168 (3)
O6—H6A···O2	0.86 (1)	1.88 (1)	2.728 (2)	170 (3)
O6—H6B···O1^ii^	0.85 (1)	2.27 (2)	3.043 (2)	152 (2)
O6—H6B···O3^ii^	0.85 (1)	2.54 (2)	3.226 (2)	139 (2)

Symmetry codes: (i) −*x*+1, *y*+1/2, −*z*+3/2; (ii) *x*−1, *y*, *z*.
